# Impact of Mild Head Injury on Neuropsychological Performance in Healthy Older Adults: Longitudinal Assessment in the AIBL Cohort

**DOI:** 10.3389/fnagi.2016.00105

**Published:** 2016-05-12

**Authors:** Matthew A. Albrecht, Colin L. Masters, David Ames, Jonathan K. Foster, The AIBL Research Group

**Affiliations:** ^1^School of Public Health, Curtin UniversityPerth, WA, Australia; ^2^Curtin Health Innovation Research Institute – BiosciencesPerth, WA, Australia; ^3^Department of Psychiatry, Maryland Psychiatric Research Center, University of Maryland School of MedicineBaltimore, MD, USA; ^4^Mental Health Research Institute, The University of MelbourneParkville, VIC, Australia; ^5^Department of Psychiatry, Academic Unit for Psychiatry of Old Age, The University of Melbourne, St. Vincent’s Aged Psychiatry Service, St. George’s HospitalParkville, VIC, Australia; ^6^National Ageing Research Institute, Royal Melbourne HospitalParkville, VIC, Australia; ^7^School of Psychology and Speech Pathology, Curtin UniversityPerth, WA, Australia; ^8^Health Department of WA, Neurosciences UnitPerth, WA, Australia

**Keywords:** mild traumatic brain injury, longitudinal, ageing, cognition, episodic memory, Bayesian

## Abstract

Traumatic brain injury (TBI) is suggested to be a significant risk factor for dementia. However, little research has been conducted into long-term neuropsychological outcomes after head trauma. Participants from the Australian Imaging, Biomarkers and Lifestyle Study of Ageing (AIBL) who had recovered after sustaining a mild TBI involving loss of consciousness more than 5 years previously were compared with matched controls across a 3-year period. Bayesian nested-domain modeling was used to estimate the effect of TBI on neuropsychological performance. There was no evidence for a chronic effect of mild TBI on any neuropsychological domain compared to controls. Within the TBI group, there was some evidence suggesting that the age that the head trauma occurred and the duration of unconsciousness were modulators of episodic memory. However, these findings were not robust. Taken together, these findings indicate that adults who have sustained a TBI resulting in loss of consciousness, but who recover to a healthy level of cognitive functioning, do not experience frank deficits in cognitive ability.

## Introduction

Traumatic brain injury (TBI) has been suggested to be a significant risk factor for the incidence of dementia (Heyman et al., 1984; Graves et al., 1990; Mortimer et al., 1991; Fleminger et al., 2003; Sivanandam and Thakur, 2012) or the earlier onset of dementia (Nemetz et al., 1999). Specifically, many studies have shown a greater incidence of Alzheimer’s type neuropathology in individuals that have sustained a TBI^+^, including increased beta amyloid deposits (Roberts et al., 1994; Uryu et al., 2007). However, the impact of TBI as a significant risk factor for Alzheimer’s disease (AD) has not always been reliably demonstrated (Shalat et al., 1987; Chandra et al., 1989; Mehta et al., 1999).

The impact of a relatively mild TBI (duration of loss of consciousness of less than 30 min; Bernstein, 1999) has been reliably associated with a variety of acute signs and symptoms, including neuropsychological impairment (Levin et al., 1987; Riggio and Wong, 2009). However, neuropsychological performance following a mild TBI (Dikmen et al., 1986; Levin et al., 1987; Schretlen and Shapiro, 2003; McCrea et al., 2009), including sports related concussions (McCrea et al., 2003; Bleiberg et al., 2004), typically returns to baseline between several days to 1 year post-injury. As such, the long-term impact of mild TBI has generally not been associated with chronic, clinically significant cognitive impairment (Frencham et al., 2005; Vanderploeg et al., 2005). However, not all research into the impact of a mild TBI has indicated that there is a lack of long-term cognitive impairment. Indeed, it has been suggested that cognitive deficits may persist longer than several years post-injury in both individuals who self-report a TBI (Carlsson et al., 1987; Klein et al., 1996; Monti et al., 2013), in individuals who sustained a sports related concussion (Beaumont et al., 2009) and in individuals who had previously been clinically examined at the time of injury (Himanen et al., 2006, 2009; Isoniemi et al., 2006; Ponsford et al., 2008; Konrad et al., 2011). For example, Klein et al. (1996) investigated the long-term effect (on average, 30 years post-injury) of a self-reported mild TBI on cognitive performance (compared with age-, sex-, and education-matched controls). Head injured participants (TBI^+^s) were found to be more impaired on a visual verbal learning task than controls on learning trials, delayed recall trials and recognition trials when they were tested several years after the event. There were also increases in reading time across all components of the Stroop task, but there was no difference between the TBI^+^ and TBI^−^ groups on the Stroop interference parameter. The authors suggested that this indicates a generalized, long-term reduction in information processing capacity in TBI^+^ participants. The cognitive differences reported by Klein et al. (1996) were found despite good clinical recovery and no ongoing subjective complaints from TBI^+^ participants. Several further studies reporting long-term cognitive contrasts between TBI^+^ and TBI^−^ individuals have yielded significant outcomes, albeit with respect to performance on a range of different tasks, including electrophysiological and functional imaging measures. Specifically, deficits have been noted on finger tapping and reaction time (Carlsson et al., 1987), auditory verbal learning (Ponsford et al., 2008; Konrad et al., 2011), visual episodic memory (Beaumont et al., 2009), digit span (Himanen et al., 2006; Ponsford et al., 2008; Konrad et al., 2011), fluency (Himanen et al., 2009; Konrad et al., 2011), go/no-go (Konrad et al., 2011), trail making (Himanen et al., 2006; Isoniemi et al., 2006; Konrad et al., 2011), digit symbol coding (Himanen et al., 2006; Ponsford et al., 2008), P300 (Beaumont et al., 2009), movement velocity (Beaumont et al., 2009), medial temporal activation during a relational memory task (Ford et al., 2013) and relational memory (Monti et al., 2013). Furthermore, some of these deficits have been linked to reduced hippocampal volumes in TBI^+^ individuals (Monti et al., 2013).

Extant research in this field therefore suggests persisting subtle cognitive deficits in TBI^+^ individuals. However, the largest study to date that has investigated the long-term effects of TBI on neuropsychological performance found no differences on many of the same or similar neuropsychological tasks that have previously revealed deficits in TBI^+^ individuals. Specifically, in their sample of 254 TBI^+^ individuals and 3214 TBI^−^ individuals, Vanderploeg et al. (2005) found no difference between TBI^+^ and TBI^−^ individuals on the Rey Complex Figure Test (RCFT), California Verbal Learning Test (CVLT) or the Delis-Kaplan Executive Function System (D-KEFS) animal fluency test. Furthermore, prospective controlled studies have generally demonstrated a full cognitive recovery after a mild head injury (Dikmen et al., 1986; Levin et al., 1987; Schretlen and Shapiro, 2003; McCrea et al., 2009) in contrast to the between groups comparison studies outlined above that have identified long-term cognitive impairments in participants who have been followed-up retrospectively. The null findings of Vanderploeg et al. (2005) also raise the question of whether sampling variability (and possibly, publication bias) may have influenced the findings of other studies. It is also the case that where some research has shown neurophysiological or cognitive impairments, they have not shown frank cognitive deficits. For example, Ford et al. (2013) were not able to demonstrate relational memory deficits despite showing alterations in neural activation patterns, and Beaumont et al. (2009) were not able to show deficits on all components of the RCFT (only on the recognition component).

There is therefore ongoing uncertainty concerning whether TBI confers and increased risk of chronic cognitive deficits. A related issue concerns whether TBI^+^ individuals are at increased risk of age-related cognitive decline. This particular question has been markedly less studied in the literature, as fewer studies have been able to access longitudinal cohorts in order to address this issue. However, it is a question of central relevance when considering the potentially increased risk of dementia with age in TBI^+^ individuals. Moreover, longitudinal investigations in older individuals also offer a potentially more powerful approach in which each individual can be treated as their own control at baseline. We were here able to examine this question in the Australian Imaging Biomarkers and Lifestyle (AIBL) cohort. In one previous longitudinal study, Himanen et al. (2006) demonstrated both cognitive enhancements and reductions in cognitive performance 30 years after the TBI^+^ group’s initial testing. However, there was no TBI^−^ control group reported in this study that had completed the baseline assessment; therefore, sampling variability was again a potential issue in this study as it is unclear whether a comparable TBI^−^ group would have displayed a similar pattern of cognitive performance over time.

Given the uncertainties arising from the existing literature, the aim of the current investigation was to investigate the AIBL cohort of over 1000 total participants to determine the effect that mild TBI resulting in loss of consciousness has on long-term neuropsychological capacity and change over time in older adults (>60 years). These are central questions of relevance when considering the potentially increased risk of dementia in TBI^+^ individuals. We hypothesized that TBI^+^ individuals would show both baseline neurocognitive deficits and more exaggerated longitudinal decline compared to controls on an extensive range of neuropsychological tests. We also undertook exploratory analyses to determine whether any deficits in performance that were observed in TBI^+^ individuals would be associated with plausibly salient features of their TBI (i.e., severity of injury, age at which the injury was sustained).

## Materials and Methods

### Participants

Participants with a history of prior TBI were identified from the AIBL cohort according to those individuals who answered “yes” to the question “Have you ever suffered a serious head injury?” during the initial AIBL screening. AIBL screening, recruitment, diagnostic classification and testing procedures are described in detail elsewhere (Ellis et al., 2009). Briefly, participants were recruited via media appeal or by their treating physician. A brief telephone screening interview took place that included questions on demographic data, medical diagnoses, perceived memory function and symptoms of depression. Individuals were excluded from the AIBL study if they reported a history of non-AD dementia, schizophrenia, bipolar disorder, Parkinson’s disease (PD) or cancer. They were also excluded if they manifested signs/symptoms of depression, symptomatic stroke, uncontrolled diabetes or regular alcohol use exceeding two standard drinks per day for women and four drinks per day for men. Participants were assigned to the following categories based upon performance on the neuropsychological battery administered and the final recommendations of a multi-disciplinary expert consensus panel: (i) Healthy Control; (ii) Mild Cognitive Impairment (MCI); or (iii) Alzheimer’s disease (AD). Below, we cover in more detail the inclusion criteria into the Healthy Control and MCI group, as individuals with MCI (or) AD at baseline were excluded from the AIBL study.

Further Healthy Control inclusion criteria were applied as per Ellis et al. (2009) and Foster et al. (2013). Healthy controls who met any of the following criteria were further discussed by the clinical review panel to confirm baseline diagnostic category: Mini Mental State Examination (MMSE) score less than 28, failure on the Logical Memory test, poor performance during neuropsychological testing (e.g., a score less than 1.5 SD below the age adjusted mean on a neuropsychological test), Clinical Dementia Rating (CDR) scale score greater than 0.5, history of any other illness likely to impede cognitive function, an informant or personal history suggestive of impaired cognitive function, or taking cognition- affecting substances at the time of the evaluation (Ellis et al., 2009).

MCI inclusion criteria were applied as per Ellis et al. (2009) and Foster et al. (2013). Participants classified as having MCI reported memory difficulties and if they were referred to the study with a provisional classification of MCI made by a clinician, demonstrated at least one neuropsychological test score which was 1.5 SD below the age-adjusted mean. If a participant volunteered to the study as a Healthy Control, to be classified as having MCI it was required that for these participants at least two of their neuropsychological test scores fell 1.5 SD below the age-adjusted mean. The magnitude of performance deficits in the individuals with MCI within the AIBL data set is on a par with other studies recruiting individuals with MCI (e.g., Petersen et al., 1999; Geslani et al., 2005; Kramer et al., 2006; Ribeiro et al., 2007; Nutter-Upham et al., 2008).

Individuals who indicated that they had received a TBI were contacted by mail approximately 18–36 months after baseline entry into AIBL to determine their interest in a follow-up study on TBI and ageing. After consent was provided to participate in the current study a structured phone interview was conducted that was consistent across all participants. This thorough interview incorporated questions regarding how the TBI occurred, what year it occurred, the duration of unconsciousness, medical attention received, whether participants attended hospital at the time of the injury (and how long they were admitted) and score on the Glasgow Coma Scale. Individuals included in this study who indicated that they had experienced a TBI demonstrated consistency in their self-report of a TBI over 18–36 months, during which time the occurrence and nature of their TBI was confirmed twice for all participants. The reliability of the approach used for characterizing TBI in the current study has been confirmed by previous studies reported in this literature (Carlsson et al., 1987; Klein et al., 1996; Monti et al., 2013).

From 74 participants with a history of TBI that were contactable from the AIBL cohort, it was confirmed for this study that 61 of these individuals had experienced at least one TBI that resulted in loss of consciousness. The remaining 13 TBI^+^ participants had received only minor head injuries not resulting in loss of consciousness or they indicated upon clarification that they had not in fact previously suffered a TBI. Of the remaining 61 TBI^+^ participants, eight individuals were classified as having MCI or AD, leaving 53 TBI^+^ Healthy Control participants who were included in the final analysis that is presented here. The duration of loss of consciousness among these TBI^+^ participants was highly skewed. The median duration of loss of consciousness was 10 min; 38 TBI^+^ individuals in the study experienced loss of consciousness lasting less than 60 min with 47 participants experiencing loss of consciousness lasting less than 1 day, leaving six participants who received a head injury with a period of unconsciousness lasting longer than several days. Due to the large range in the duration of loss consciousness and the highly skewed distribution, duration of loss of consciousness was log transformed before analysis. TBI^+^ participants were closely matched with 104 TBI^−^ participants (i.e., two TBI^−^ participants for each TBI^+^ participant where possible, in order to increase statistical power) from the AIBL database on the following variables: age, sex, Wechsler Test of Adult Reading (WTAR) score, APOE status and education. All TBI^+^ and TBI^−^ participants in the current study were categorized as Healthy Controls at baseline according to the AIBL clinical consensus criteria (see Ellis et al., 2009).

Participants were tested three times: at baseline, 18 months and 36 months. Attrition rates are shown in “Supplementary Table 2”.

### Ethics

Ethical approval for the AIBL study was obtained in Victoria and Western Australia from the St. Vincent’s Hospital, Austin Health, Edith Cowan University and Hollywood Private Hospital Human Research Ethics Committees. Approval for the TBI component of the study was obtained from Curtin University’s human research ethics committee. Written informed consent was obtained from all participants. The research was completed in accordance with the Declaration of Helsinki.

### Cognitive Assessment

The battery of cognitive tests that was administered to participants included the MMSE, CVLT second edition (CVLT-II), Logical Memory I and II from the Wechsler Memory Scale (WMS; Story A only), D-KEFS verbal fluency subtests (letter fluency, category fluency and category switching), 30-item Boston Naming Test (BNT), WTAR, Digit Span and Digit-Symbol Coding subtests of the Wechsler Adult Intelligence Scale-third edition (WAIS-III), Stroop test (Victoria version) and the RCFT.

### Apolipoprotein E Genotyping

Participants provided an 80 mL blood sample shortly after arrival for the first AIBL assessment session. Full details of the blood analysis that was conducted in this sample is reported in Ellis et al. (2009). APOE genotype was determined by direct sequencing.

### Statistical Analysis

A Bayesian nested domain hierarchical modeling approach was used, similar to the method outlined in Thurston et al. (2009). The nested domain model takes into account the relationships between multiple outcome measures that are nested within domains (e.g., one of the key measures used to evaluate the domain of Verbal Episodic Memory was the CVLT, which comprises several different but correlated outcome measurements). The nested domain model was used because approaches that separately fit each outcome variable as a function of the exposure variable (plus covariates) do not benefit from the overlap in information shared amongst variables. This can result in reduced stability of effect estimates and a reduction in power to detect an effect of the exposure variable (Thurston et al., 2009). The increase of power and stability of the nested domain model is conferred by its hierarchical basis which has been shown to reduce Type S (sign) and Type M (magnitude) errors by partially pooling estimates of outcomes within domains towards each other (Gelman et al., 2012). The approach has been described by Woodard et al. (2013; p. 786) as a “…type of continuous latent factor model…”, and is similar to other types of multivariate analyses including structural equation modeling and latent variable analysis. Latent variable types of analyses model the effect of an exposure variable on a set of unobserved latent variables that are formed from the observed outcome variables. By contrast, the nested domain approach models the effects of the exposure variable on the outcomes directly, but pools effects with a high correlation towards each other using random effects (Woodard et al., 2013). This can be thought of as an extended version of the mixed model approach, which is more robust to violations of the latent variable assumptions; e.g., bias due to a subset of uncorrelated outcomes being associated with the exposure variable and lack of robustness due to the presence of covariance parameters in the mean (Sammel et al., 1999). The nested domain approach can be easily extended to be more robust to other deviations from an ideal model (e.g., robust to outliers) by using a t-distribution to model the residuals (Lange et al., 1989; Kruschke, 2013). It can also be extended to take into account longitudinal designs by fitting both “random” intercepts and slopes for each person and for each person for each cognitive domain. We adopted this approach here.

The cognitive domains (and associated outcome measures) assessed in the present study were as follows: Primary Memory (D1—total digits forward and backward), Perceptual Speed (D2—Digit-Symbol Coding, Stroop “Words”, “Dots”), Verbal Episodic Memory (D3—logical memory I and II, CVLT total learning trials, short-delay free recall, long-delay free recall, recognition “hits”, signal detection “d-prime”), Visuospatial Functions (D4—RCFT copy, copy time), Visual Episodic Memory (D5—RCFT 3 min delay, 30 min delay and recognition), Verbal Ability (D6—BNT and D-KEFS letter fluency, category fluency, and switching fluency) and Interference (D7—Stroop “Colours” and interference parameter [“Colors”/“Dots”]). The selection of outcome measurements to be nested within each domain were chosen *a priori*, based upon: (1) the instruments that were administered to participants in the AIBL study; (2) the cognitive domain that a series of outcome measures is considered to evaluate; and (3) the perceptual modality of the episodic memory items (as visual and verbal episodic memory possess some separable neurological underpinnings).

Each outcome measure was modeled as a function of TBI status (presence or absence), time since entry into the study (baseline, 18 and 36 months) and with respect to the interaction between TBI status and time. In addition, TBI severity (measured by duration of unconsciousness) and the age that the earliest TBI occurred in each individual were also investigated within the TBI^+^ group. For these final analyses, age at baseline, WTAR IQ and sex were included as covariates in order to control for these factors. Numeric variables were scaled to a mean = 0 and SD = 1. Where appropriate, variables were inverted so that larger scores indicated better performance across neuropsychological measures (e.g., Stroop). The scaling of all variables consequently meant that the parameter estimates are similar to partial correlation coefficients (Thurston et al., 2009).

An objective approach to setting the priors was used; i.e., priors could be described as weakly informative, centered around an effect of zero. Priors for the overarching parameters were described by a normal distribution centered on zero and with SD = 100 (relatively flat for standardized variables). The priors for all domain level parameters and outcome level parameters (nested within domains) were described by normal distributions, centered on zero and with a SD estimated from a half-Cauchy distribution centered on zero and scale equal to 1 (Gelman and Hill, 2007). The priors for the random effects parameters were similarly estimated; however, the SD was estimated from a uniform distribution bounded between 0 and 10. The SD prior for the outcome level errors of the *t*-distributions were uniform between 0 and 10. The degrees of freedom prior used for each outcome was described by the inverse of a uniform distribution bounded between 0.001 and 0.5. A large estimate for the degrees of freedom parameter indicates that the residuals can be described by a normal distribution, while a smaller degrees of freedom parameter indicates that the data have fatter tails and data points in this region are appropriately down-weighted. For the supplementary exploratory analyses, measures were not grouped into domains and a diffuse prior for the outcome level effects was described by a normal distribution centered on zero and SD = 1000.

Fifty thousand Markov Chain Monte Carlo (MCMC) samples, thinned every 5th step, were taken for each model. Convergence and auto-correlation were monitored using the Gelman-Rubin statistic (Gelman and Rubin, 1992) and by ensuring that more than 1000 effective samples for all parameters of interest were taken. Posterior means and 95% highest density intervals (HDIs; Kruschke, 2011) are given for each of the parameters.

Statistical analysis was performed in R version 3.0 using the “rjags” package to interface with the Gibbs sampler “JAGS” version 3.3.0 (Plummer, 2003, 2011). All observed data were included in each analysis.

## Results

### Demographics

Table 1 presents the demographic variables for the TBI^+^ and the TBI^−^ groups, together with the TBI characteristics for the TBI^+^ group. There was little evidence for demographic differences between the two groups, particularly on the variables that informed the matching of TBI^+^ and TBI^−^ individuals (age, sex, education, premorbid IQ, and APOE ε4 genotype). Means (+SD) for each neuropsychological measure for each time point across TBI^+^ and TBI^−^ participants are provided in Supplementary Table 1 and missing data tabulations for each measure are presented in Supplementary Table 2.

**Table 1:** **Demographic and TBI characteristics**.

	TBI^−^	TBI^+^	*p*
**Demographic variables**
Age (years, mean + SD)	70.2 (5.9)	70.2 (5.5)	0.97
Sex (F/M)	43/61	22/31	1.00
Education (7-12/13-15/15 +/NA)	38/14/52	19/8/25/1	0.89
APOE ε4 alleles (0/1/2)	70/30/4	35/16/2	1.00
Retired (No/Yes)	26/78	7/46	0.13
Relationship status (Partnered/Separated/Widowed/Single)	87/4/7/6	35/7/7/4	0.07
Birth Place (Australia/UK/Other/NA)	79/14/11	37/13/2/1	0.10
Primary language (English/Other/NA)	102/2	52/0/1	0.80
Number of sessions at time of analysis (1/2/3)	0/15/89	0/1/52	0.02
**Cognitive State**
Mini mental state exam (mean + SD)	29.0 (1.1)	28.9 (1.1)	0.58
WTAR IQ (mean + SD)	110.0 (6.3)	110.6 (6.7)	0.56
Clinical dementia rating (0/0.5)	100/4	50/3	0.91
CDR sum of boxes (0/0.5/1)	98/5/1	50/2/1	0.85
HADS depression (mean + SD)	2.4 (2.2)	2.7 (2.7)	0.55
HADS anxiety (mean + SD)	4.0 (2.8)	3.6 (3.0)	0.46
**Head injury variables**
Longest length of loss of consciousness (min, median + range)	10 (0.03–30240)
Number of people with HIs <30 min/>30 min	35/18
Age at earliest HI (years, median + range)	18 (4–67)
Obtained medical attention in a hospital (Y/N)	26/27
Duration of stay in hospital at least 1 day or overnight (Y/N)	22/4
Number of HIs (1/2/3)	40/10/3

### The Effect of Incidence of TBI on Cognitive Performance and Age-Related Decline

**Figure 1:**
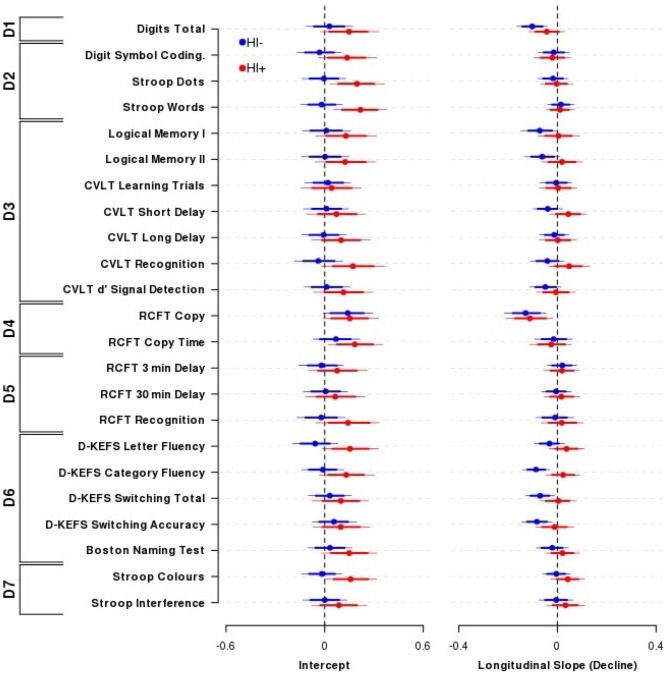
**Effect of Traumatic brain injury (TBI) on cognitive performance.** The influence of TBI on neuropsychological performance across outcomes and domains comparing Head Injury absent participants (TBI^−^, blue circles and lines) with Head Injury present participants (TBI^+^, red circles and lines). Displayed are the mean (±80, 95% highest density intervals (HDIs)) parameter estimates from the nested domain model; these are equivalent to partial correlation coefficients. Individual parameter estimates are shrunk towards each other within domains, and domains are shrunk towards a common estimate. Positive indicates better performance (Intercept) or improvement in performance over time (Longitudinal slope). Overall, there was little difference in cognitive performance (left) or decline (right) between TBI^+^ or TBI^−^ participants. *N* = 102 TBI^−^ and 52 TBI^+^ participants. Domains: D1—Primary Memory, D2—Perceptual Speed, D3—Verbal Memory, D4—Visuospatial Function, D5—Visual Memory, D6—Verbal Ability, D7—Interference.

Figure 1 presents the intercept and slope parameter estimates (± 80, 95% HDIs) from the Bayesian nested domain regression for the TBI^−^ and TBI^+^ groups. Parameter estimates from a domain agnostic model are given in Supplementary Figure 1.

Overall performance and age-related cognitive change for the TBI^+^ group compared to the TBI^−^ group was similar across the range of neuropsychological tests administered. The outcome measures within the Primary Memory (D1), Perceptual Speed (D2), Verbal Memory (D3), Visuospatial Function (D4), Visual Memory (D5), Verbal Ability (D6) and Interference (D7) domains all possessed substantially overlapping credible intervals for the TBI^+^ and TBI^−^ groups. The outcome measures that demonstrated the largest group effect sizes for baseline performance were CVLT recognition (TBI^+^-TBI^−^ contrast = 0.21, 95% HDI = −0.03, 0.46), D-KEFS letter fluency (0.21, 95% HDI = −0.01, 0.44) and Stroop Words (TBI^+^-TBI^−^ contrast = 0.24, 95% HDI = 0.02, 0.45), Colours (0.17, 95% HDI = −0.02, 0.38) and Dots (0.20, 95% HDI = −0.02, 0.42). However, it is important to note that the credible intervals of these contrasts included zero, or if they did not include zero they were nevertheless unsupportive of the experimental hypothesis.

By contrast, the outcome measures that demonstrated the largest effect sizes for differential age-related cognitive decline (i.e., time by TBI interaction) were CVLT recognition (TBI^+^-TBI^−^ slope contrast = 0.09, 95% HDI = −0.02, 0.20), CVLT short-delay recall (0.08, 95% HDI = −0.01, 0.18) and D-KEFS category fluency (0.11, 95% HDI = 0.02, 0.20). However, it is important to note that again the credible intervals of these contrasts included zero or if they did not they were nevertheless unsupportive of the experimental hypothesis.

### The Influence of Severity and Age of Head Injury on Cognitive Performance

**Figure 2:**
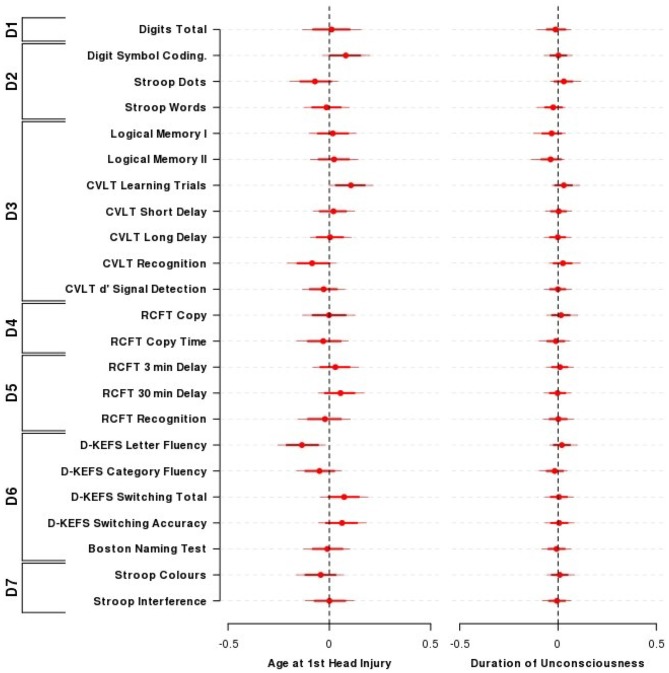
**Effect of the age of TBI and duration of unconsciousness on cognitive performance.** The influence of the age at first TBI and the duration of loss of consciousness on neuropsychological performance across outcomes and domains among individuals who had previously experienced a TBI. Presented are the mean (±80, 95% HDI) parameter estimates for the nested domain model that included age at first TBI, duration of loss of consciousness, time since entry into the study along with the covariates age, sex, and wechsler test of adult reading (WTAR). Parameter estimates are equivalent to partial correlation coefficients. *N* = 52. Domains: D1—Primary Memory, D2—Perceptual Speed, D3—Verbal Memory, D4—Visuospatial Function, D5—Visual Memory, D6—Verbal Ability, D7—Interference.

Figure 2 presents the results from the Bayesian nested domain regression which modeled the effects of: (i) TBI severity (measured by taking the log_10_ of the longest period of unconsciousness a person experienced); and (ii) the earliest age that a TBI occurred. Only participants who identified positively as TBI (i.e., TBI^+^) cases were included in this analysis. Parameter estimates from a domain agnostic model that did not include covariates are given in Supplementary Figure 2.

All 95% credible intervals for the influence of TBI severity contained zero, indicating that a longer duration of unconsciousness was not substantially associated with reduced cognitive performance in this sample. Logical Memory I and II showed the largest effect sizes for duration of loss of consciousness (LMI = −0.03, 95% HDI = −0.13, 0.04; LMII = −0.04, 95% HDI = −0.14, 0.03). An exploratory approach that did not take into account the relationships between and within cognitive domains indicated that the credible intervals for both logical memory I and II excluded zero (LMI = −0.26, 95% HDI = −0.48, −0.03; LMII = −0.29, 95% HDI = −0.52, −0.06; Figure 2).

The majority of coefficients from the nested domain model (Figure 2) did not indicate a substantial association between the age that TBI occurred and neuropsychological performance. Performance on the D-KEFS letter fluency demonstrated the largest effect size and a credible interval that only just excluded zero (D-KEFS letter fluency = −0.14, 95% HDI = −0.26, −0.02). Using an exploratory approach as above (i.e., without taking into account relationships between and within cognitive domains; Supplementary Figure 2), there was evidence for sparing of verbal episodic memory performance for each year older a person was when their TBI occurred (CVLT sum of learning trials = 0.32, 95% HDI = 0.10, 0.55; CVLT short delay = 0.24, 95% HDI = 0.01, 0.48).

## Discussion

There has been inconsistent identification in previous reports of long-term cognitive deficits (i.e., >1 year post-injury) of individuals of who have previously sustained a mild TBI. Several research groups have found persistent deficits in a number of cognitive domains and neurophysiological indices (Carlsson et al., 1987; Klein et al., 1996; Himanen et al., 2006, 2009; Isoniemi et al., 2006; Ponsford et al., 2008; Beaumont et al., 2009; Konrad et al., 2011; Broglio et al., 2012; Ford et al., 2013; Monti et al., 2013). However, the largest study to date that has investigated the long-term effects of TBI on neuropsychological performance found no reliable long-term deficits in TBI^+^ individuals (Vanderploeg et al., 2005). The present analyses indicated that in a large cohort that was followed longitudinally over 3 years TBI^+^ individuals demonstrated no significant neuropsychological deficits compared to matched TBI^−^ individuals, consistent with the findings reported by Vanderploeg et al. (2005) who evaluated performance on some of the same neuropsychological measures that were used in the current study. In addition, there was no indication in the present study of increased cognitive decline in TBI^+^ participants over time when compared to matched TBI^−^ individuals.

The current findings suggests that individuals who have sustained a mild TBI and whose post-injury cognitive performance has stabilized are not likely to show more pronounced deterioration of cognitive functioning as they age, when compared to non-head injured individuals. However, most individuals in this study had suffered what would generally be categorized as a mild TBI, such that the current findings should not necessarily be generalized to individuals who have sustained a more severe TBI but have nevertheless survived into their sixth decade of life.

In combination with the findings of Vanderploeg et al. (2005), the present results suggest that there is minimal overall long-term neuropsychological consequence of a mild TBI on cognitive performance when individuals who have sustained such an injury are compared with non-injured, matched controls. The current findings further suggest that older individuals who have sustained a TBI several years ago and who have recovered to manifest a level of overall healthy functioning do not appear to be susceptible to increased cognitive decline after their sixth decade of life, as measured over a period of 36 months. However, a longer follow-up period would provide more definitive information concerning the possibility of accelerated age-related cognitive decline and increased risk of dementia following mild TBI earlier in life. In addition, Broglio et al. (2012) suggest that individuals with a high level of cognitive reserve may be relatively spared from the impact of TBI on age-related cognitive decline. Given that the mean estimated IQ in the present article was 110, our TBI^+^ participants may possess enough cognitive reserve to withstand the potentially deleterious impact of TBI on age-related cognitive decline. Again, a longer follow-up period may have revealed differences between the TBI^+^ and the TBI^−^ groups, even in relatively high functioning individuals.

Despite the lack of an overall negative effect of TBI on cognitive performance observed in the present study, the age that TBI occurred and the severity of TBI were identified as potentially important modulators of neuropsychological performance in exploratory analyses that were undertaken. Specifically, verbal episodic memory (as measured by the CVLT) was associated with better outcomes if the age that the TBI occurred was delayed until later in life. The majority of articles that have evaluated long-term neuropsychological outcome following TBI (i.e., >5 years) in older adults did not report a significant effect of age of injury on cognitive performance (Vanderploeg et al., 2005; Himanen et al., 2006, 2009; Isoniemi et al., 2006; Konrad et al., 2011). Where such an analysis was undertaken, the direction of the effect was unclear (Himanen et al., 2006), or failed to reach statistical significance (Carlsson et al., 1987). Given the inconsistent results in the literature to date, the present results pointing towards a link between episodic memory and the age that the TBI occurred should be treated somewhat cautiously (especially considering that this effect emerged using a less conservative and more exploratory statistical approach). At the same time, it is worth noting that decline in episodic memory is considered a hallmark of early AD, which is one of the principal age-related neurological conditions associated with previous TBI (e.g., Heyman et al., 1984; Plassman et al., 2000; Magnoni and Brody, 2010; Sivanandam and Thakur, 2012). In addition, the recent finding reported by Monti et al. (2013) of bilateral hippocampal reductions in TBI^+^ participants (who, similarly, sustained a TBI more than several years prior to testing) compared with TBI^−^ participants is consistent with the episodic memory findings obtained using the supplementary, exploratory analyses that are reported here. The hippocampus is one of the key brain regions implicated in subserving episodic memory (Squire, 1992; Chadwick et al., 2010; Ranganath, 2010; Eichenbaum et al., 2012).

The index of severity used in the current study (duration of loss of consciousness) was not reliably associated with performance on any of the composite cognitive domains. However, performance on the Logical Memory tests (I and II) demonstrated the largest effect size, and under less conservative statistical conditions performance on these measures did demonstrate an association with TBI severity. Himanen et al. (2006) and Levin and Eisenberg (1979) both found that increased severity of TBI was associated with poorer outcomes on measures of verbal episodic memory in adults and children respectively. The findings in the present study, when considered in the context of those of Himanen et al. (2006) and Levin and Eisenberg (1979), suggest that verbal episodic memory capacity may be at particular risk of being adversely affected after a more severe TBI, consistent with a putative increased risk of developing AD in individuals who have experienced a significant TBI. These considerations notwithstanding, it should be borne in mind that no overall group effect of TBI on any of the measures of episodic memory was observed in this study.

The evidence presented in this article has some limitations. Firstly, records of participant head injuries were obtained retrospectively and without reference to hospital records or third person verification. As such, information obtained on the age, duration and severity of head injury may be somewhat imprecise. Furthermore, the initial screening question used a single question to identify participants for follow-up assessment, which has been shown by some researchers to underestimate the incidence of a TBI (Diamond et al., 2007; Bogner and Corrigan, 2009). Indeed, the proportion of participants in the AIBL cohort reporting a significant TBI appears to be below the prevalence estimates reported in the literature (Corrigan et al., 2010; Whiteneck et al., 2016). However, the self-reported nature of the description of TBI used in the present article is similar to that used in several previous published studies, including well cited studies in the field (Carlsson et al., 1987; Klein et al., 1996; Vanderploeg et al., 2005; Monti et al., 2013). Moreover, participants in the current study were asked about their incidence of TBI at the time of entry to the main AIBL study and again at the time of recruitment into the present study, approximately 4 years later. Therefore, a degree of consistency in reporting a history of TBI over a period of 18–36 months was required before inclusion of data in the present study. Nevertheless, the self-report nature of the duration of unconsciousness variable could result in both under and over estimation of the length of unconsciousness without external validation. Less precision for this variable may result in a reduced ability to detect associations with duration of unconsciousness. However, this would not significantly influence the major findings of the present article estimating the overall effect of a mTBI on cognitive performance. Secondly, we did not have information available in this study on individuals who unfortunately did not survive after a TBI, although it is unlikely that overall mortality rates would be high when considering the types of mild TBI that were the focus of the present investigation. A prospective study would be able to address this issue definitively.

A considerable strength of the present study is application of a robust Bayesian analysis method that explicitly took into account the relationships amongst neuropsychological measures, while at the same time presenting a more commonly used mixed-model approach (see Supplementary Figures 1 and 2). Furthermore, the nested-domain method incorporates more information into each parameter estimate, protecting against potentially inflated effects by shrinking estimates towards each other within each domain (Thurston et al., 2009).

The current study found that adults who have sustained a TBI resulting in loss of consciousness, but who recover to a healthy level of cognitive functioning, do not experience frank deficits in cognitive ability. However, under less conservative statistical conditions specific chronic associations with verbal episodic memory capacity were indicated. This may be a potentially significant consideration with respect to age-related decline in episodic memory, which is an important early sign of late onset dementia. In addition, our participants were cognitively healthy at entry into the study and the follow-up period was for 36 months, potentially limiting sensitivity to any possible enhancement of neurodegeneration in our TBI sample. Further investigation is warranted in other large, longitudinal cohorts of aging individuals.

## Author Contributions

All authors listed, have made substantial, direct and intellectual contribution to the work, and approved it for publication.

## Conflict of Interest Statement

The authors declare that the research was conducted in the absence of any commercial or financial relationships that could be construed as a potential conflict of interest.
