# Supplementary material for: Impact of Mild Head Injury on Neuropsychological Performance in Healthy Older Adults: Longitudinal Assessment in the AIBL Cohort
Source: Front Aging Neurosci. 2016 May 12;8:105. doi: 10.3389/fnagi.2016.00105 (PMC4863889; doi:10.3389/fnagi.2016.00105)
Supplement: Supplementary file 1 [file Image_1.pdf]

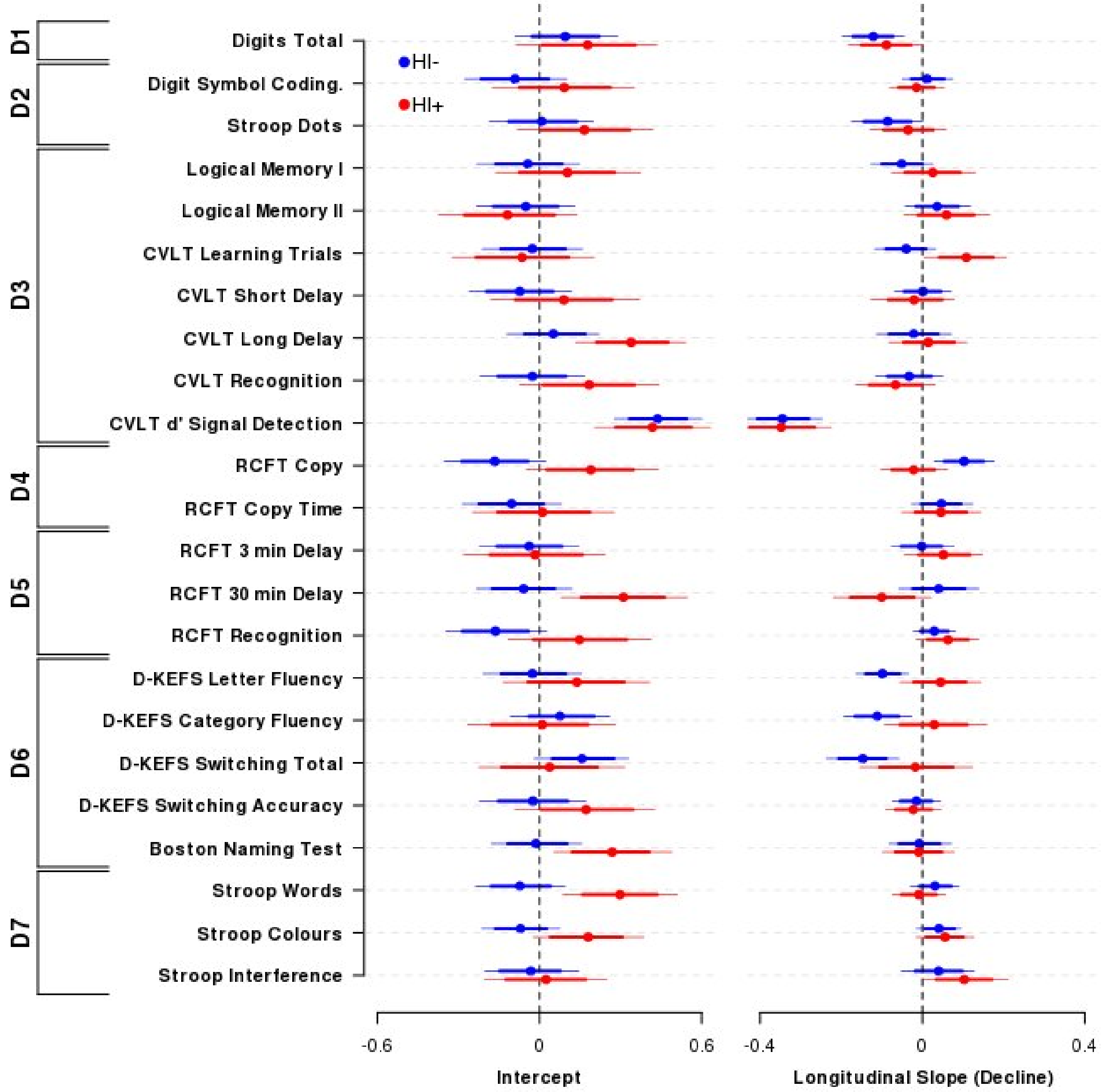

Supplementary Figure 1 – Unadjusted coefficients for the effect of head injury on cognitive performance and cognitive decline (see also Figure 1): The influence of head injury on neuropsychological performance across outcomes and domains comparing Head Injury absent participants (HI^-, blue circles and lines) with Head Injury present participants (HI^+, red circles and lines). Displayed are the mean (± 80, 95% HDI) parameter estimates from a robust mixed-effects model (with varying intercepts and varying slopes per participant). This model does not incorporate the shrinkage of the model depicted in Figure 1. N = 102 HI^- and 52 HI^+ participants.

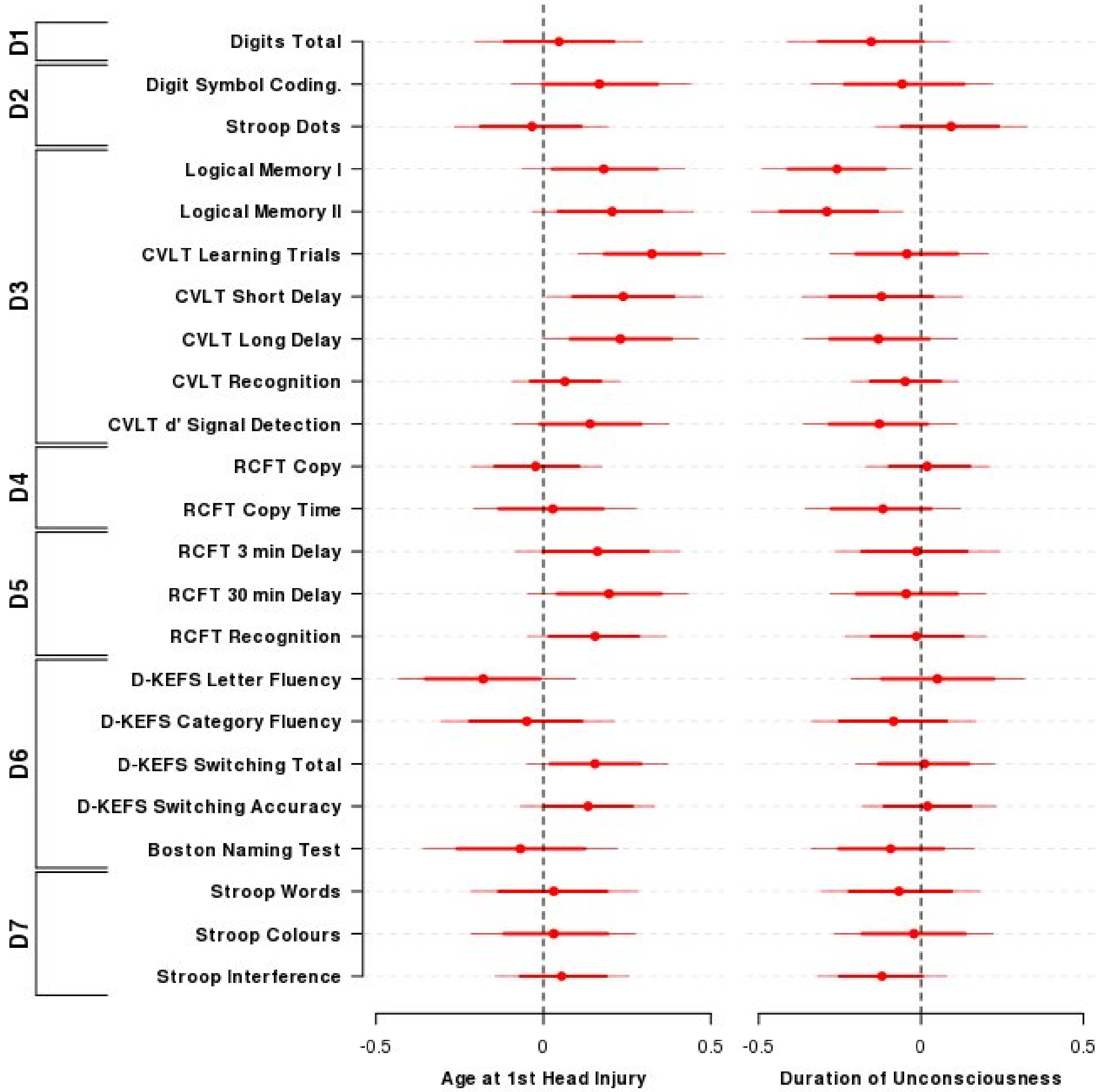

Supplementary Figure 2 – Unadjusted coefficients for the effect of age of HI and duration of unconsciousness on cognitive performance (see also Figure 2): The influence of the Age at first head injury and the duration of loss of consciousness on Neuropsychological performance across outcomes in the group that had previously experienced a head injury. Presented are the mean (± 80, 95% HDI) parameter estimates from separate robust mixed-effects model for age of loss of consciousness and duration of unconsciousness respectively (with varying intercepts and varying slopes per participant) while excluding the covariates that were used in Figure 2. This model does not incorporate the shrinkage of the model depicted in Figure 2. N = 52.
